# Web-based questionnaire survey for exploring engagement characteristics of advance care planning in Japan: a cross-sectional study

**DOI:** 10.1186/s13104-024-06699-7

**Published:** 2024-02-08

**Authors:** Yasuhiro Nakanishi, Yukio Tsugihashi, Akira Hayasaka, Yuichi Nishioka, Manabu Akahane

**Affiliations:** 1 Department of Health and Welfare Services, National Institute of Public Health, 2-3-6 Minami, 351-0197 Wako, Saitama Japan; 2 Department of Public Health, Health Management and Policy, Nara Medical University, 840 Shijo-Cho, 634-8521 Kashihara, Nara Japan; 3 Mathematics and Informatics, Osaka Kyoiku University, 4-698-1 Asahigaoka, 582-8582 Kashiwara, Osaka Japan

**Keywords:** Advance care planning, Cardiopulmonary resuscitation, Public health policies, Medical professionals, Caregivers, Japan, Cross-sectional study, Web-based questionnaire, Survey

## Abstract

**Objective:**

Definitive promotion of advance care planning (ACP) practices will require policy interventions tailored to the characteristics of the Japanese population and society. However, effective policies for promoting ACP are currently lacking in Japan. This study aimed to explore the characteristics of Japanese people who engaged in ACP activities through a web-based questionnaire survey, which was administered to individuals aged 25–64 years and classified into four occupational categories (non-medical/non-caregiving professionals [general population], physicians, nurses, and caregivers).

**Results:**

The total sample size was 1,648, with equal occupational category and age group distributions. Respondents in the general population group were less likely to discuss or document ACP than those in the other groups. Stepwise logistic regression analysis showed a significant difference in the adjusted odds ratio (aOR) of the independent variables of “attended cardiopulmonary resuscitation (CPR) training session(s)” (aOR: 1.93; 95% confidence interval [CI]: 1.18–3.15) and “having experience in performing CPR” (aOR: 2.61; 95% CI: 1.51–4.54) for respondents who discussed ACP with their families. A significant difference was observed in the aOR of the independent variable of “having experience in performing CPR” (aOR: 4.58; 95% CI: 2.30–9.13) for respondents who documented a written record of ACP.

**Supplementary Information:**

The online version contains supplementary material available at 10.1186/s13104-024-06699-7.

## Introduction

Globally, a large number of studies exploring advance care planning (ACP) have been conducted in recent years [1, 2]. ACP is a crucial tool for facilitating decisions regarding future medical care preferences, and is associated with beneficial outcomes for patients, healthcare professionals, and the healthcare system, including improved documentation of end-of-life discussions and preferences, as well as healthcare cost reductions in certain situations for particular demographics [2]. A recent scoping review regarding ACP trials indicated that, although intervention effects for goal concordance and quality of life outcomes were mixed, written-only interventions, interactive, multimedia/multimodal programs, facilitated discussions, video-only interventions, and clinician training were predominantly positive [1].

Among developed countries, Japan has the fastest aging society, with 28.6% of the population aged 65 years or older as of 2020 [3], and the longest average life expectancy as well as healthy life expectancy [4]. However, after reaching a peak of approximately 128 million in 2008, its population has been steadily declining since 2011 [5]. While this decline is expected to continue, the number of deaths in Japan, which has been steadily increasing every year, reached 1,142,407 in 2008 [6], and is expected to increase by approximately 1.47 times by 2040 [7]. In March 2018, the Ministry of Health, Labour and Welfare (MHLW) revised the Guidelines on the Decision-making Process for End-of-Life Care, adding a new provision recommending ACP. This revision was made due to the growing demand for home medical care, long-term care, and end-of-life care support due to the aging society with high mortality rates, and to address the necessity of developing a nationwide community-based integrated care system. The revision’s background was also the dissemination of research and initiatives based on the concept of ACP, mainly in Western countries [8, 9]. Although the importance of ACP has been recognized and the national policy in Japan supports its implementation [8], the prevalence of ACP practices among the general population and medical and long-term care professionals is lower than that in Western countries, such as the United States and Canada [10–13]. A nationwide questionnaire survey on the engagement in ACP activities among the general population, including medical and long-term care workers, was conducted in December 2017 by the MHLW [10]; however, the characteristics of individuals with engagement in ACP activities, which could help formulate an effective policy in further promoting ACP, are still unknown. In particular, only a few studies have reported on the characteristics of individuals who engaged in ACP activities in the general Japanese population [14–16].

Previous investigations have shown that the influence of life events, facing not only one’s illness but also the illness or death of a close relative, might act as a catalyst for ACP engagement [10, 17, 18]. The life event may also affect one’s end-of-life care arrangements and preferences, and those influences may be more strongly discerned through informal discussions, rather than formal ACP conversations [17]. Previous studies have also shown that educational and decision support programs using videos on cardiopulmonary resuscitation (CPR) effectively promote ACP [19–22].

When promoting ACP practices, it is important to consider the target population’s national characteristics and cultural aspects. Although Delphi studies of Western countries have established a consensus for ACP practices [23, 24], their findings may not apply to Asian countries due to their distinctive, family-oriented cultures [25–28]. Japan has specific values and family relationships, which highlight the harmony between patients and families. Conversely, Western countries, where ACP is developed, are focused on an individual’s right to self-determination. Thus, it is important to engage in ACP activities in the context of Eastern cultures with respect for the harmony between patients and families, in a patient-centered decision-making support system [9, 29].

Thus, policy interventions that have been adapted according to the Japanese society are needed for additional promotion of ACP practices, which Japan currently lacks. Therefore, we used a web-based questionnaire survey with an aim to explore the characteristics of Japanese people with engagement in ACP activities.

## Main text

### Methods

#### Survey design and participants

A cross-sectional, web-based, questionnaire survey was conducted in Japan through a large internet research agency (Macromill Inc.), which had approximately 1.2 million qualified panelists in 2019. A number of clinical and public health studies have been previously conducted using the internet surveys of this research agency [30–33]. The participants of this study included registered panel members aged 25–64 years. First, the survey agency created a list using random sampling across all registrants to recruit participants. Next, an email for assessing interest in survey participation was sent to all individuals on the list. The respondents were divided into 10-year age groups (25–34, 35–44, 45–54, and 55–64 years) across four occupational categories, including non-medical/non-caregiving professionals (general population), physicians, nurses, and caregivers (certified care workers and care managers). Registration was stopped when the number of respondents in each group reached the target sample size (100 respondents in each of the 16 groups). The study participants provided their informed consent online before anonymously responding to the survey. They received an explanation of the procedure prior to the beginning of the survey and were informed that they could interrupt or terminate the survey without providing a reason. The questionnaire survey was administered to the participants from December 13 to 15, 2019.

#### Survey of engagement in ACP activities

A preliminary survey, acting as a pilot test, was conducted in a set of 15 individuals (approximately 1% of the total sample size for this questionnaire) to evaluate completeness, clarity, and consistency. Adjustments were made to the survey questionnaire based on the findings from the pilot test. This pilot test aimed to identify any inconsistencies or discrepancies in the survey items and make necessary adjustments to ensure the overall coherence of the questionnaire.

Parameters such as gender, age, marital status, having/not having a child or children, yearly income, and region (eight areas of Hokkaido, Tohoku, Kanto, Chubu, Kinki, Chugoku, Shikoku, and Kyushu) were pre-recorded during the members’ registration to the survey panel. The survey inquired about their engagement in ACP activities, attending cardiopulmonary resuscitation (CPR) training sessions, and performing CPR.

In the survey, the participants were requested to answer the questions Q3 (“Have you ever had discussions with your family about your preferred future treatment or care?”) and Q4 (“Do you have any written documentation on the content of your discussions about your preferred future treatment or care?”) to assess their experience in the aforementioned practices. Additionally, the participants were asked Q5 (“Have you ever had discussions with your grandparents’ and parents’ (including in-laws) about their preferred future treatment or care?”) and Q6 (“Do you have any written documentation on the content of your grandparents’ and parents’ [including in-laws] discussions about their preferred future treatment or care?”). Furthermore, we asked the respondents about their participation in CPR training sessions (chest compressions and artificial respiration [mouth-to-mouth] techniques) and the application of an automated external defibrillator (AED). Participants were asked to respond to each question using a six-point Likert scale as follows: 1: Strongly disagree (or strongly no), 2: Disagree (or no), 3: Somewhat disagree (or somewhat no), 4: Somewhat agree (or somewhat yes), 5: Agree (or yes), 6: Strongly agree (or strongly yes) (as shown in Additional File 1).

### *Statistical analysis*

We categorized the data into two binary values: “disagree” or “no” (which included 1: Strongly disagree [or strongly no], 2: Disagree [or no], and 3: Somewhat disagree [or somewhat no]) and “agree” or “yes” (which included 4: Somewhat agree [or somewhat yes], 5: Agree [or yes], 6: Strongly agree [or strongly yes]). Respondents who answered “yes” to question Q2 (“Please tell us about your participation in resuscitation technique classes.”) and indicated Q2S1 (“I have learned chest compression techniques”), Q2S2 (“I have learned artificial respiration [mouth-to-mouth] techniques.”), or Q2S3 (“I have learned how to use an automated external defibrillator [AED].”) were considered as having attended CPR training session(s). Respondents who answered “yes” to the question Q1 (“Have you ever found someone who suddenly collapsed and performed the following?”) and indicated Q1S3 (“I have performed chest compressions.”), Q1S4 (“I have performed artificial respiration [mouth-to-mouth].”), or Q1S5 (“I have used an automated external defibrillator [AED].”) were considered as having actual experience in performing CPR (as shown in Additional File 1). Differences in engagement in ACP activities across different age groups and occupational categories were compared using the chi-square test (two-tailed). The dataset for the general population group was weighted by gender and age using demographic data from the 2015 census [34]. Next, the population corresponding to the medical and other health services industries category was excluded from the overall Japanese population to calculate the weighting coefficients. In addition, forward stepwise logistic regression analysis was performed for the weighted general population group using the respondents’ experience in discussing or documenting future treatment and care preferences for themselves, their grandparents, and their parents as a dependent variable. In the model, experience in attending CPR training session(s) and actual experience in performing CPR were retained (forced) as independent variables. Demographic variables such as gender, age (four categories), marital status, having/not having a child or children, and yearly income (five categories) were also included as independent variables using the stepwise method.

As a sub-analysis, forward stepwise logistic regression analysis was also conducted for the entire study cohort, which included respondents from four occupational groups. This analysis used the respondents’ experience in discussing or documenting future treatment and care preferences for themselves, their grandparents, and their parents as a dependent variable. Experience in attending CPR training sessions and actual experience in performing CPR were forcibly included as independent variables. Variables that showed a significant association in the stepwise logistic regression analysis conducted specifically for the weighted general population group were also forced into the model. In addition, the respondents’ occupation was also forced as a covariate in the model. It is important to note that the entire study cohort could not be weighted using demographic data from the census. Since the census data do not contain gender- and age-specific data for each profession such as physicians, nurses, caregivers, etc., it is not possible to calculate weighting coefficients for each occupation. This limitation could potentially introduce a bias that is not present in the general population. Therefore, we performed the logistic regression analysis primarily for the weighted general population group. The sub-analysis for the entire cohort was performed as a reference to examine the differences compared with the analysis results for the weighted general population group.

All statistical analyses were performed using IBM SPSS Statistics for Windows, version 27.0 (IBM Corp., Armonk, NY, United States), with the level of statistical significance set at *p* < 0.05.

## Results

The characteristics of the respondents categorized according to age group and occupations are summarized in Table 1. The total sample size was 1,648, with equal occupational category and age group distributions. A high proportion of respondents, excluding the general population, attended CPR training session(s). As for their experience in performing CPR, higher percentages of physicians and nurses answered having had the experience compared with general population.

**Table 1:** **Characteristics of respondents**

Characteristics		All *N* = 1,648		General *N* = 412		Physician *N* = 412		Nurse *N* = 412		Caregiver *N* = 412	
		N	%	N	%	N	%	N	%	N	%
Women		969	58.8	242	58.7	113	27.4	359	87.1	255	61.9
Age (years)	25–34	412	25.0	103	25.0	103	25.0	103	25.0	103	25.0
	35–44	412	25.0	103	25.0	103	25.0	103	25.0	103	25.0
	45–54	412	25.0	103	25.0	103	25.0	103	25.0	103	25.0
	55–64	412	25.0	103	25.0	103	25.0	103	25.0	103	25.0
Married		1,072	65.0	257	62.4	299	72.6	269	65.3	247	60.0
Having child/children		1,019	61.8	225	54.6	262	63.6	269	65.3	263	63.8
Income level per year (million yen)	0–1.99	282	17.1	133	32.3	7	1.7	68	16.5	74	18.0
	2–3.99	407	24.7	98	23.8	25	6.1	99	24.0	185	44.9
	4–5.99	255	15.5	48	11.7	36	8.7	114	27.7	57	13.8
	Greater than 6	380	23.1	45	10.9	277	67.2	46	11.2	12	2.9
	No answer/Do not know	324	19.7	88	21.4	67	16.3	85	20.6	84	20.4
Region (Eight areas)	Hokkaido	123	7.5	25	6.1	30	7.3	38	9.2	30	7.3
	Tohoku	109	6.6	35	8.5	25	6.1	20	4.9	29	7.0
	Kanto	417	25.3	116	28.2	101	24.5	95	23.1	105	25.5
	Chubu	302	18.3	74	18.0	74	18.0	71	17.2	83	20.1
	Kinki	310	18.8	74	18.0	90	21.8	77	18.7	69	16.7
	Chugoku	114	6.9	29	7.0	26	6.3	31	7.5	28	6.8
	Shikoku	47	2.9	13	3.2	12	2.9	15	3.6	7	1.7
	Kyushu	226	13.7	46	11.2	54	13.1	65	15.8	61	14.8
Attended CPR training session(s)		1,440	87.4	279	67.7	392	95.1	400	97.1	369	89.6
Having experience in performing CPR		799	48.5	65	15.8	280	68.0	280	68.0	174	42.2

CPR: cardiopulmonary resuscitation

Note: The approximate average exchange rate in 2019 was 110 yen to 1 US dollar [35]

Figure S1 (Additional File 2) shows the percentage of respondents who had experience in discussing and documenting their future treatment and care. The findings of our analysis indicated that, among the four age groups, respondents in the 55–64 years age group had significantly higher rates of discussing their future treatment and care preferences with their families. Meanwhile, those in the 25–34 years age group showed significantly lower rates of such discussions within the same groups. In terms of occupational categories, nurses and caregivers had significantly higher rates of discussing their preferences with their families in the four occupational categories. Conversely, the general population showed significantly lower rates of such discussions within these categories. In addition, although no significant differences were observed among the age groups, the percentage of respondents who had a written record of such preferences significantly tended to be higher for caregivers and lower for general population in the four occupational categories.

Figure S2 (Additional File 3) shows the percentage of respondents who had experience in discussing and documenting the future treatment and care of their grandparents and parents. Although no significant differences were observed among age groups, caregivers had significantly higher rates of discussing the future treatment and care preferences of their grandparents and parents in the four occupational categories, and general population had significantly lower rates of such discussions within these categories. Similarly, the percentage of respondents who documented such preferences was significantly higher for caregivers and lower for general population in the four occupational groups. However, significant differences were not observed in the age groups.

Table 2 shows the results of stepwise logistic regression analysis of respondents’ experience in discussing or documenting their future care targeted for the weighted general population group (*N* = 412). The characteristics of the weighted general population group are shown in Table S1 (Additional File 4). The number of respondents who discussed and documented a written record of their own future treatment and care preferences was 145 (35.2%) and 46 (11.2%), respectively. Respondents who had attended CPR training were more likely to have discussed their future care. Similarly, respondents with experience in performing CPR were more likely to have discussed their future care and were more likely to have documented it. Respondents in the 55–64 age group were more likely to have discussed their future care compared with respondents in the 25–34 age group.

**Table 2:** Stepwise logistic regression analysis of respondents’ experience in discussing or documenting their future care (weighted general population group, *N* = 412)

Independent variables		Discussed			Documented		
		aOR	95% CI	*p*-value	aOR	95% CI	*p*-value
Attended CPR training session(s)		1.93	1.18–3.15	0.008	1.22	0.57–2.61	0.606
Having experience in performing CPR		2.61	1.51–4.54	< 0.001	4.58	2.30–9.13	< 0.001
Age (years)	25–34	Ref					
	35–44	1.25	0.67–2.32	0.490			
	45–54	1.45	0.77–2.71	0.247			
	55–64	2.41	1.25–4.65	0.009			

aOR: adjusted odds ratio, Ref: reference, CI: confidence interval, CPR: cardiopulmonary resuscitation

Table 3 shows the results of stepwise logistic regression analysis of respondents’ experience in discussing or documenting their grandparents’ and parents’ future care targeted for the weighted general population group (*N* = 412). The number of respondents who discussed and documented a written record of their grandparents’ or parents’ future treatment and care preferences was 141 (34.1%) and 38 (9.3%), respectively. Respondents who had attended CPR training were more likely to have discussed their grandparents’ and parents’ future care and were more likely to have documented it. Respondents with experience in performing CPR were more likely to have documented their grandparents’ and parents’ future care. Women were less likely than men to have documented their grandparents’ and parents’ future care.

**Table 3:** Stepwise logistic regression analysis of respondents’ experience in discussing or documenting their grandparents’ and parents’ future care (weighted general population group, *N* = 412)

Independent variables	Discussed			Documented		
	aOR	95% CI	*p*-value	aOR	95% CI	*p*-value
Attended CPR training session(s)	2.86	1.74–4.67	< 0.001	7.58	1.92–29.95	0.004
Having experience in performing CPR	1.55	0.90–2.67	0.112	2.90	1.40–5.99	0.004
Women				0.44	0.21–0.93	0.032

aOR: adjusted odds ratio, CI: confidence interval, CPR: cardiopulmonary resuscitation

Table 4 shows the results of stepwise logistic regression analysis of respondents’ experience in discussing or documenting their future care targeted for the entire study cohort (*N* = 1,648). The number of respondents who discussed and documented a written record of their own future treatment and care preferences was 703 (42.7%) and 177 (10.7%), respectively. Similar to the results of the analysis for the weighted general population group, respondents who had attended CPR training were more likely to have discussed their future care. Respondents with experience in performing CPR were more likely to have discussed their future care and were more likely to have documented it. Respondents in the 45–54 and 55–64 age groups were more likely to have discussed their future care compared with respondents in the 25–34 age group.

**Table 4:** Stepwise logistic regression analysis of respondents’ experience in discussing or documenting their future care (targeted for entire study cohort, *N* = 1,648)

Independent variables		Discussed			Documented			
		aOR	95% CI	*p*-value	aOR	95% CI	*p*-value	
Attended CPR training session(s)		1.81	1.27–2.58	0.001	0.81	0.46–1.41	0.458	
Having experience in performing CPR		1.86	1.48–2.33	< 0.001	3.04	2.07–4.45	< 0.001	
Age (years)	25–34	Ref						
	35–44	1.08	0.81–1.44	0.580				
	45–54	1.47	1.11–1.96	0.008				
	55–64	1.67	1.25–2.23	< 0.001				
Occupations	General	Ref			Ref			
	Physician	0.83	0.60–1.14	0.246	0.71	0.42–1.19		0.193
	Nurse	1.14	0.83–1.56	0.425	0.58	0.34–1.00		0.048
	Caregiver	1.38	1.03–1.86	0.033	1.40	0.88–2.23		0.156

aOR: adjusted odds ratio, Ref: reference, CI: confidence interval, CPR: cardiopulmonary resuscitation

Table 5 shows the results of stepwise logistic regression analysis of respondents’ experience in discussing or documenting their grandparents’ and parents’ future care targeted for the entire study cohort (*N* = 1,648). The number of respondents who discussed and documented a written record of their grandparents’ or parents’ future treatment and care preferences was 700 (42.5%) and 162 (9.8%), respectively. Respondents who had attended CPR training were more likely to have discussed their grandparents’ and parents’ future care. Respondents with experience in performing CPR were more likely to have documented their grandparents’ and parents’ future care and were more likely to have documented it. Compared to the results of the analysis for the weighted general population group, there were no considerable differences in the overall trends, although the results differed slightly, with no significant differences by gender.

**Table 5:** Stepwise logistic regression analysis of respondents’ experience in discussing or documenting their grandparents’ and parents’ future care (targeted for entire study cohort, *N* = 1,648)

Independent variables		Discussed			Documented			
		aOR	95% CI	*p*-value	aOR	95% CI	*p*-value	
Attended CPR training session(s)		2.12	1.49–3.03	< 0.001	1.42	0.74–2.72	0.293	
Having experience in performing CPR		1.45	1.16–1.81	0.001	2.08	1.42–3.04	< 0.001	
Women					0.98	0.68–1.41	0.909	
Occupations	General	Ref			Ref			
	Physician	0.95	0.70–1.30	0.757	0.94	0.55–1.62		0.829
	Nurse	1.00	0.73–1.37	0.993	0.67	0.38–1.19		0.170
	Caregiver	1.53	1.14–2.05	0.005	1.52	0.93–2.49		0.092

aOR: adjusted odds ratio, Ref: reference, CI: confidence interval, CPR: cardiopulmonary resuscitation

## Discussion

Our study showed a statistically significant trend toward the general population being less likely than healthcare and caregiving professionals to discuss or document a written record of the future treatment and care preferences for themselves, their grandparents, or their parents. Furthermore, the results of the stepwise logistic regression analysis revealed that attending CPR training sessions and having actual experience in performing CPR were associated with engaging in ACP activities. To the best of our knowledge, this is the first study to report such findings among the general Japanese population.

The willingness to engage in ACP among medical workers differs depending on their occupation [36], and these differences are also seen in their efforts for themselves [37]. A recent study in the United States [37] compared the use of ACP components such as living wills, durable power of attorney for healthcare, and informal discussions among individuals working in medical professions (e.g., physicians and nurses), legal professions (e.g., lawyers), social/health services professions (e.g., clergy and social workers), other professions (e.g., teachers and engineers), and other non-professional occupations (e.g., secretaries or laborers). The study findings indicated that medical professionals were significantly more likely to have discussed their end-of-life preferences than other professional workers, and non-professionals such as secretaries or laborers had significantly lower trends for discussing their preferences than other professionals. However, our findings showed that the percentage of respondents who discussed their future treatment and care preferences with their families tended to be higher among caregivers than among physicians, while it was similar in nurses and caregivers. Caregivers in Japan may be more likely to encounter older adults who need ACP, compared to general medical professionals, as their daily work requires being in contact with residents of nursing homes and users of long-term care services. Regarding the lower tendency of the general population to engage in ACP activities, one factor causing this could be different levels of awareness about ACP between the general population and medical and caregiving professionals. According to a nationwide questionnaire survey in Japan, the percentage of respondents who answered that they were “unaware” of ACP was 75.5% among the general population compared to 41.6–51.6% among medical and caregiving professionals [10]. The lower level of awareness about ACP among the general population might be a contributing factor to their lesser engagement in ACP activities. Another potential factor could be differences in professional experience compared to medical and caregiving professionals. Compared to physicians, nurses, and caregivers, the general population typically have fewer opportunities to actually participate in ACP activities or to recognize the necessity of ACP. Medical professionals, with their clinical experience and formal training, have greater knowledge of end-of-life treatments and the complexities of decision-making than the general public. Additionally, caregiving professionals often have more frequent interactions with older adults compared to the general population. Therefore, there is a need for opportunities to provide more information and raise awareness about ACP among the general population.

In terms of the association between age and engagement in ACP activities, our analysis showed that there was a significant trend for respondents to have more discussions about their own future care as they aged. This result is consistent with previous research [12, 14], and may be attributed to the possibility that, as people age, they become more interested in how they will face the end of their life.

Regarding the relationship between engagement in ACP activities and gender, previous studies in Western countries, such as the United States and Canada, have shown a significant tendency for women to have more experience in discussions about ACP [12, 38]. However, previous studies targeting the general population in Japan have not found a significant association between ACP discussions and gender, which aligns with our study results [15, 16]. Furthermore, in our analysis focusing on the weighted general population group, women were significantly less likely to document their grandparents’ and parents’ future care. A previous study [38] using data from the Wisconsin Longitudinal Study indicated that women were less likely than men to engage in formal planning only, such as a living will or a durable power of attorney for health care. However, our study found no significant gender difference in documenting one’s own future care, which suggests that this aspect may require further investigation in the future.

A randomized controlled trial in the United States of America [20] examined the impact of a video decision support tool and patient checklist on ACP implementation, including content on CPR or intubation, for patients with heart failure. The results showed that patients who had undergone video-assisted intervention were more knowledgeable about their care options, more likely to prefer medical care focused on quality of life and comfort, and more likely to forgo invasive interventions than patients in the verbal control group. Another pilot RCT in the USA [19], which focused on the impact of an educational CPR video on participants with progressive exocrine pancreas and hepatobiliary cancers, demonstrated a statistical trend toward more ACP documentation (advance directive or documented discussions with medical providers) in the post-test month compared to a similar CPR narrative. Understanding and decision-making in CPR are important components of ACP. Previous studies have shown that a better understanding of CPR may facilitate ACP implementation [19−22]. In this respect, in Japan, CPR training is provided to the general population mainly by the municipal fire departments and the Japanese Red Cross Society [39, 40]. The number of people who attended the CPR training session held by the fire departments was around two million, one year before the coronavirus disease (COVID-19) pandemic [41]. Moreover, a CPR course is required by Japanese people to obtain a driver’s license. Children and older students are taught CPR at school [39, 42]. In 1994, basic life support education became compulsory and has since been included in primary, junior high, and high school curricula [40]. More than 90% of schools encourage and provide CPR instruction for students along with training for teachers and staff. Additionally, most schools have at least one AED on-site [43, 44]. Previous investigations have shown that the influence of life events, such as facing not only one’s illness but also the illness or death of a close relative, might act as a catalyst for ACP engagement [10, 17, 18]. We regarded the CPR training and education received by many Japanese people, either in schools or as part of licensing processes, as a proxy for experiencing life events, and explored the potential relationship between these experiences and engagement in ACP discussions and documentation. Considering that opportunities for the general population to attend CPR training sessions are becoming more available in Japan, one effective approach could be, for example, linking ACP awareness-raising initiatives with CPR training sessions as a measure of increasing ACP awareness among the general population. By doing so, further promoting CPR training could become one of the strategies for advancing ACP, potentially leading to an effective dissemination strategy for ACP among the general population. This study serves as an initial exploration, highlighting the need for further detailed research to understand the feasibility and implications of such an approach in the context of Japan’s distinctive cultural and healthcare setting.

## Conclusions

The results of this study indicated that the engagement rate of the general population in ACP activities is lower than that of medical and caregiving professionals and that attending CPR training sessions and experiences performing CPR are associated with engagement in ACP activities. One potentially effective approach to improve the ACP engagement rate among the general population would be to link ACP awareness with CPR training sessions.

### Limitations

This study has some limitations. First, the data were self-reported, and no respondent behavior was actually observed. Second, the respondents in the study were limited to internet users, and thus, caution should be exercised in interpreting the findings of the analysis. However, it is worth noting that in Japan, the percentage of internet users is remarkably high [45]. Third, the analysis of physicians and nurses categorized by occupation in this study did not take into account the details of their specialty. Therefore, the results may differ if physicians and nurses who specialize in end-of-life care, such as palliative care, are included. Fourth, this study was conducted with a focus on policy perspectives to explore the characteristics of Japanese peoples’ engagement in ACP activities, and the questions asked in our web-based survey regarding engagement in ACP activities specifically focused on preferred future treatment or care for oneself as well as for grandparents and parents. The definition of ACP as inferred from the questions could be more narrowly focused than those of values, goals, and preferences [23].

### Electronic supplementary material

Below is the link to the electronic supplementary material.

Additional File 1: Textual information: Survey questions included in this study

Additional File 2: Figure S1: Respondents with experience discussing and documenting personal future treatment and care preferences

Additional File 3: Figure S2: Respondents with experience discussing and documenting future treatment and care preferences of grandparents and parents

Additional File 4: Table S1: Characteristics of the unweighted and weighted general population groups

## Data Availability

The datasets generated and/or analyzed during the current study are not publicly available due to participant privacy, but are available from the corresponding author on reasonable request.

## Abbreviations

ACP: Advance care planning
CPR: Cardiopulmonary resuscitation
AED: Automated external defibrillator
