# Supplementary material for: Web-based questionnaire survey for exploring engagement characteristics of advance care planning in Japan: a cross-sectional study
Source: BMC Res Notes. 2024 Feb 8;17:47. doi: 10.1186/s13104-024-06699-7 (PMC10854018; doi:10.1186/s13104-024-06699-7)
Supplement: Supplementary file 1 — Additional File 1: Textual information: Survey questions included in this study [file 13104_2024_6699_MOESM1_ESM.docx]

**Additional information:** Survey questions included in this study

This questionnaire contains a section inquiring about the future treatment and care of both you and your family. If you consent to the purpose of this questionnaire, please respond to the questions. If you prefer not to respond, you can click on the “Stop Responding” button or simply close your browser to exit the survey. All survey results will be processed such that individual respondents cannot be identified. The data will be used for societal benefits and future research.

Please rate your response to each question using the following six-point scale: 1: Strongly disagree (or strongly no), 2: Disagree (or no), 3: Somewhat disagree (or somewhat no), 4: Somewhat agree (or somewhat yes), 5: Agree (or yes), 6: Strongly agree (or strongly yes).

Q1. Have you ever found someone who suddenly collapsed and performed the following?

S1. I have talked to the person who suddenly collapsed

S2. I have called an ambulance (dialing 119)

S3. I have performed chest compressions

S4. I have performed artificial respiration (mouth-to-mouth)

S5. I have used an automated external defibrillator (AED)

Q2. Please tell us about your participation in resuscitation technique classes.

S1. I have learned chest compression techniques

S2. I have learned artificial respiration (mouth-to-mouth) techniques

S3. I have learned how to use an automated external defibrillator (AED)

S4. I have learned that mortality increases rapidly in the first few minutes after cardiopulmonary arrest

S5. My knowledge is not up to date, so I would like to relearn the techniques

Q3. Have you ever had discussions with your family about your preferred future treatment or care?

Q4. Do you have any written documentation on the content of your discussions about your preferred future treatment or care?

Q5. Have you ever had discussions with your grandparents’ and parents’ (including in-laws) about their preferred future treatment or care?

Q6. Do you have any written documentation on the content of your grandparents’ and parents’ (including in-laws) discussions about their preferred future treatment or care?
