# Supplementary material for: Web-based questionnaire survey for exploring engagement characteristics of advance care planning in Japan: a cross-sectional study
Source: BMC Res Notes. 2024 Feb 8;17:47. doi: 10.1186/s13104-024-06699-7 (PMC10854018; doi:10.1186/s13104-024-06699-7)
Supplement: Supplementary file 2 — Additional File 2: Figure S1: Respondents with experience discussing and documenting personal future treatment and care preferences [file 13104_2024_6699_MOESM2_ESM.pptx]

## Slide 1
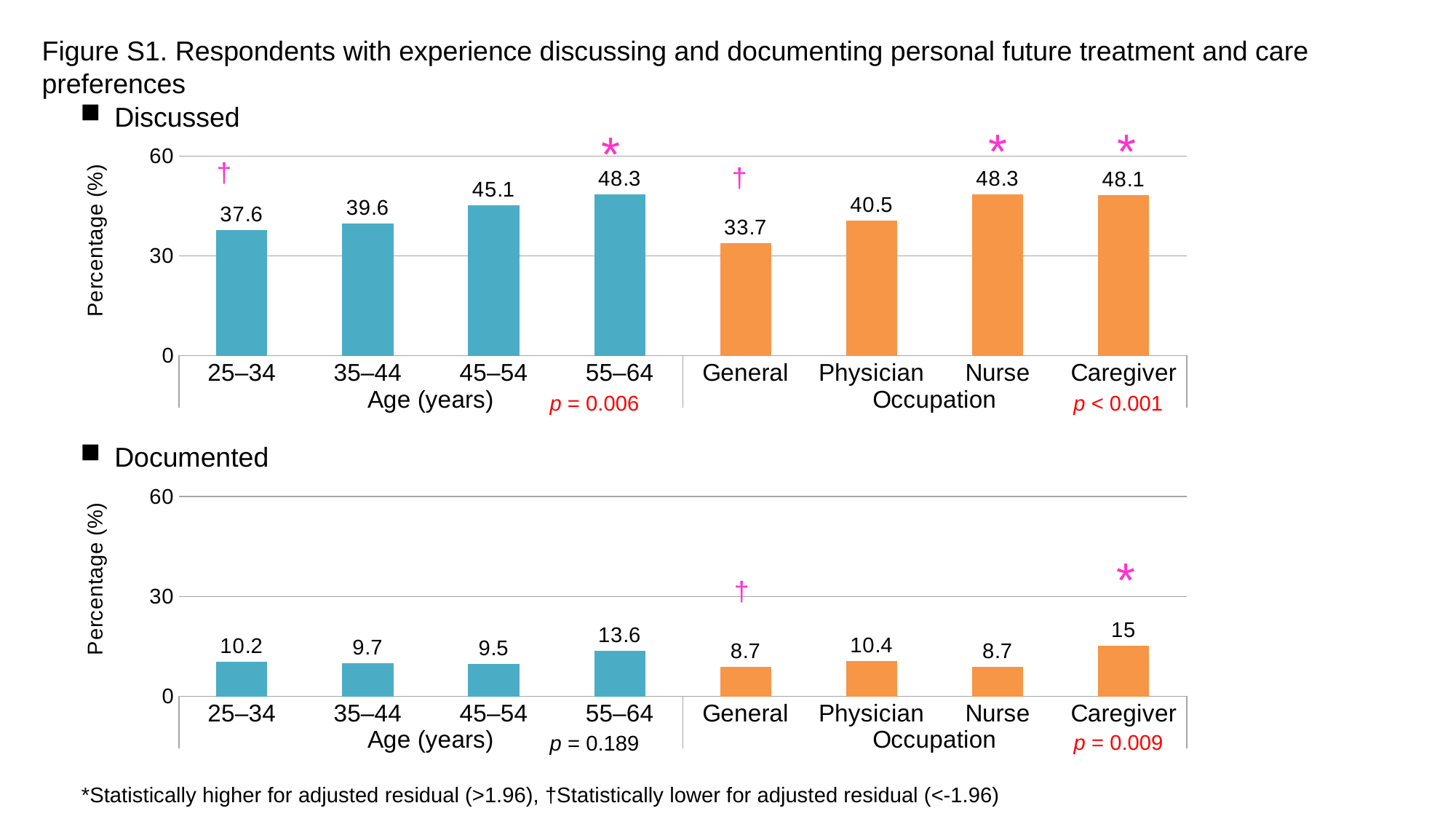

Figure S1. Respondents with experience discussing and documenting personal future treatment and care preferences
Discussed
*
*
*
### Chart
| Category | 話し合ったことがある |
|---|---|
| 25–34 | 37.6 |
| 35–44 | 39.6 |
| 45–54 | 45.1 |
| 55–64 | 48.3 |
| General | 33.7 |
| Physician | 40.5 |
| Nurse | 48.3 |
| Caregiver | 48.1 |†
†
p = 0.006
p < 0.001
Documented
### Chart
| Category | 残している |
|---|---|
| 25–34 | 10.2 |
| 35–44 | 9.700000000000001 |
| 45–54 | 9.5 |
| 55–64 | 13.600000000000001 |
| General | 8.7 |
| Physician | 10.4 |
| Nurse | 8.7 |
| Caregiver | 15.0 |*
†
p = 0.009
p = 0.189
*Statistically higher for adjusted residual (>1.96), †Statistically lower for adjusted residual (<-1.96)
