# Supplementary material for: Web-based questionnaire survey for exploring engagement characteristics of advance care planning in Japan: a cross-sectional study
Source: BMC Res Notes. 2024 Feb 8;17:47. doi: 10.1186/s13104-024-06699-7 (PMC10854018; doi:10.1186/s13104-024-06699-7)
Supplement: Supplementary file 3 — Additional File 3: Figure S2: Respondents with experience discussing and documenting future treatment and care preferences of grandparents and parents [file 13104_2024_6699_MOESM3_ESM.pptx]

## Slide 1
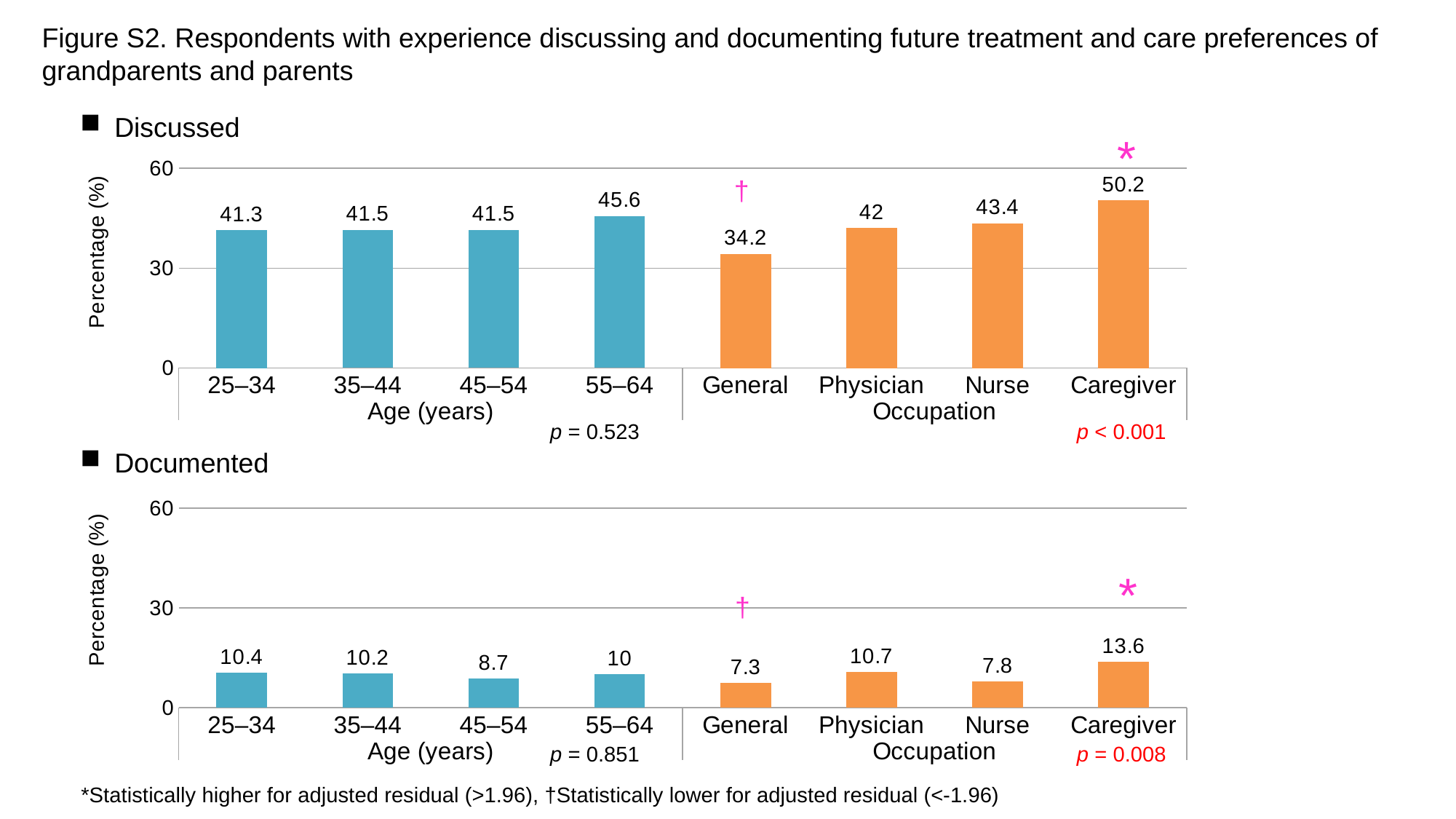

Figure S2. Respondents with experience discussing and documenting future treatment and care preferences of grandparents and parents
Discussed
*
### Chart
| Category | 話し合ったことがある |
|---|---|
| 25–34 | 41.3 |
| 35–44 | 41.5 |
| 45–54 | 41.5 |
| 55–64 | 45.6 |
| General | 34.2 |
| Physician | 42.0 |
| Nurse | 43.4 |
| Caregiver | 50.2 |†
p = 0.523
p < 0.001
Documented
### Chart
| Category | 残している |
|---|---|
| 25–34 | 10.4 |
| 35–44 | 10.2 |
| 45–54 | 8.7 |
| 55–64 | 10.0 |
| General | 7.3 |
| Physician | 10.7 |
| Nurse | 7.8 |
| Caregiver | 13.600000000000001 |*
†
p = 0.851
p = 0.008
*Statistically higher for adjusted residual (>1.96), †Statistically lower for adjusted residual (<-1.96)
