# Supplementary material for: Web-based questionnaire survey for exploring engagement characteristics of advance care planning in Japan: a cross-sectional study
Source: BMC Res Notes. 2024 Feb 8;17:47. doi: 10.1186/s13104-024-06699-7 (PMC10854018; doi:10.1186/s13104-024-06699-7)
Supplement: Supplementary file 4 — Additional File 4: Table S1: Characteristics of the unweighted and weighted general population groups [file 13104_2024_6699_MOESM4_ESM.docx]

**Table S1.** Characteristics of the unweighted and weighted general population groups

| Characteristics | | Unweighted  N = 412 | | Weighted  N = 412 | |
| --- | --- | --- | --- | --- | --- |
|  |  | N | % | N | % |
| Women | | 242 | 58.7 | 200 | 48.6 |
| Age (years) | 25–34 | 103 | 25.0 | 87 | 21.2 |
|  | 35–44 | 103 | 25.0 | 115 | 27.9 |
|  | 45–54 | 103 | 25.0 | 106 | 25.7 |
|  | 55−64 | 103 | 25.0 | 104 | 25.1 |
| Married | | 257 | 62.4 | 251 | 61.0 |
| Having child/children | | 225 | 54.6 | 226 | 54.8 |
| Income level per year (million yen) | 0–1.99 | 133 | 32.3 | 134 | 32.5 |
|  | 2–3.99 | 98 | 23.8 | 87 | 21.2 |
|  | 4–5.99 | 48 | 11.7 | 53 | 12.8 |
|  | Greater than 6 | 45 | 10.9 | 49 | 11.9 |
|  | No answer/Do not know | 88 | 21.4 | 89 | 21.6 |
| Region (eight areas) | Hokkaido | 25 | 6.1 | 28 | 6.8 |
|  | Tohoku | 35 | 8.5 | 35 | 8.5 |
|  | Kanto | 116 | 28.2 | 121 | 29.4 |
|  | Chubu | 74 | 18.0 | 68 | 16.5 |
|  | Kinki | 74 | 18.0 | 75 | 18.2 |
|  | Chugoku | 29 | 7.0 | 23 | 5.6 |
|  | Shikoku | 13 | 3.2 | 16 | 3.9 |
|  | Kyushu | 46 | 11.2 | 45 | 10.9 |
| Attended CPR training session(s) | | 279 | 67.7 | 268 | 65.1 |
| Having experience in implementing CPR | | 65 | 15.8 | 69 | 16.8 |

CPR: cardiopulmonary resuscitation

Note: The approximate average exchange rate in 2019 was 110 yen to 1 US dollar.
